# Change in the pulp chamber temperature with different stripping techniques

**DOI:** 10.1186/s40510-014-0055-8

**Published:** 2014-09-25

**Authors:** José Carlos d’Ornellas Pereira, André Weissheimer, Luciane Macedo de Menezes, Eduardo Martinelli Santayana de Lima, Maurício Mezomo

**Affiliations:** Department of Orthodontics, School of Dentistry, Centro Universitário Franciscano (UNIFRA), 97015-513 Santa Maria, Brazil; Universidade Federal do Rio Grande do Sul (UFRGS), 90040-060 Porto Alegre, Brazil; Department of Orthodontics, School of Dentistry, Pontifícia Universidade Católica-RS (PUC-RS), 90619-900 Porto Alegre, Brazil

**Keywords:** Orthodontics, Stripping, Pulp chamber, Temperature

## Abstract

**Background:**

The aim of this study is to evaluate the change in pulp chamber temperature during the stripping technique.

**Methods:**

Seventy-eight proximal surfaces of 39 extracted human teeth were stripped by two techniques: double-sided perforated stripping disk (PSD) and handheld stripper (HS). The teeth were divided into three groups: incisors (group 1), premolars (2), and molars (3). A J type thermocouple was inserted into the pulp chamber for temperature evaluation during the stripping procedure.

**Results:**

Temperature rise was observed in all groups. The average temperature increase for the incisors was 2.58°C (±0.27°C) with PSD and 1.24°C (±0.3°C) with HS; for the premolars, 2.64°C (±0.29°C) with PSD and 0.96°C (±0.39°C) with HS; and for the molars, 2.48°C (±0.38°C) with PSD and 0.92°C (±0.18°C) with HS. There was significant difference (*p* < 0.001) in pulp temperature variation among the stripping techniques evaluated. Greater variations in the temperature were observed for the stripping technique with PSD for all groups (3.1°C in incisors and premolars, 3.2°C in molars). Stripping performed with HS had minor differences in pulp temperature (1.7°C in incisors, 1.9°C in premolars, and 1.2°C in molars) than those in PSD group. However, the temperature variation was less than the critical threshold (5.5°C) in all groups. The results for teeth group comparison showed no significant difference in the temperature variation.

**Conclusions:**

The stripping technique with PSD produced significant increase in pulp temperature, with no differences between the types of teeth. However, it may not be clinically relevant, and both stripping techniques can be used safely.

## Background

The classic study by Zach and Cohen held in the teeth of primates showed that increase of 5.5°C in the pulp chamber can cause considerable damage, compromising the pulp health and producing irreversible inflammation in 40% of the specimens tested [1]. The possible detrimental effect of the increased temperature in the pulp tissue during clinical procedures has been a matter of concern in dentistry [2]. The heat transferred to the pulp can result in histopathological changes and necrosis of the pulp tissue [3].

The stripping technique is a clinical procedure characterized by the interproximal enamel reduction of the teeth. In this procedure, the created space is used for tooth alignment in cases of crowding [4] to correct the curve of spee and camouflage some malocclusions [5], enhancing dental esthetics by recontouring [6,7]. Being a procedure routinely performed in clinical orthodontics for non-extraction treatments [8], some studies evaluated the possible deleterious effects of stripping technique on the proximal area. Enamel irregularities, such as scratches, resulting from an inadequate technique could increase the susceptibility of these teeth to the accumulation of plaque with consequent higher propensity to decays and periodontal diseases [9-11]. On the other hand, Craig and Sheridan [12] and Sheridan and Ledoux [13] concluded that posterior teeth whose interproximal enamel were stripped are not more susceptible to decay or periodontal disease.

Another possible deleterious effect of stripping technique is pulp tissue damage caused by the heat generated during the process. Therefore, Zachrisson [7] and Sheridan and Ledoux [13] emphasize the need for refrigeration during the procedure. However, visibility is a key factor to perform stripping procedures in order to avoid injuries to the periodontal tissues and scaring of the proximal enamel, which makes the use of cooling a negative factor. So, to properly visualize the procedure, the use of water can be a problem.

Different techniques for the stripping procedure are recommended. Handheld strippers, diamond and carbide burs cooling at high speed, and perforated stripping disks at low speed without refrigeration are the most common [14-16]. Some factors such as the size and type of burs used, the duration of contact, use of abrasive tools, and the power employed influence the heat generation during the procedure, which can generate enough heat to permanently damage the pulp tissue [15-18].

Studies that have evaluated the change in the pulp chamber temperature during stripping procedures are scarce. Therefore, the aim of this study was to evaluate, *in vitro*, the temperature change in the pulp chamber of human incisors, premolars, and molars using metal handheld strippers and perforated stripping disks without refrigeration for the stripping procedure.

## Methods

This project was approved by the ethics committee in research of the Franciscan University Center-UNIFRA (no. 112.2011.2). For the sample size, calculation determined the alpha value of 5% and power by 80%. It was also considered that the temperature variation would be significant from a difference of 0.5° between the averages. It established the need of 13 teeth in each group for testing. From UNIFRA human teeth bank, 13 permanent human lower incisors, 13 first lower premolars, and 13 lower first molars, totaling 78 proximal surfaces, with intact dental crowns were selected. The teeth were cleaned, soaked, and stored in saline solution.

The teeth were fixed, by their root portion, to a colorless autopolymerizing acrylic resin support, included in a PVC cylinder. The dental crown remained fully exposed to the implementation of research procedures. A 2-mm diameter cavity was made with spherical diamond burs (1016, KG Sorensen, São Paulo, Brazil), from the lingual surface for the incisors and the occlusal surface for premolars and molars, following the pulp chamber. Pulp tissue debris were removed with a manual curette and irrigated with sodium hypochlorite at 1%.

**Figure 1:**
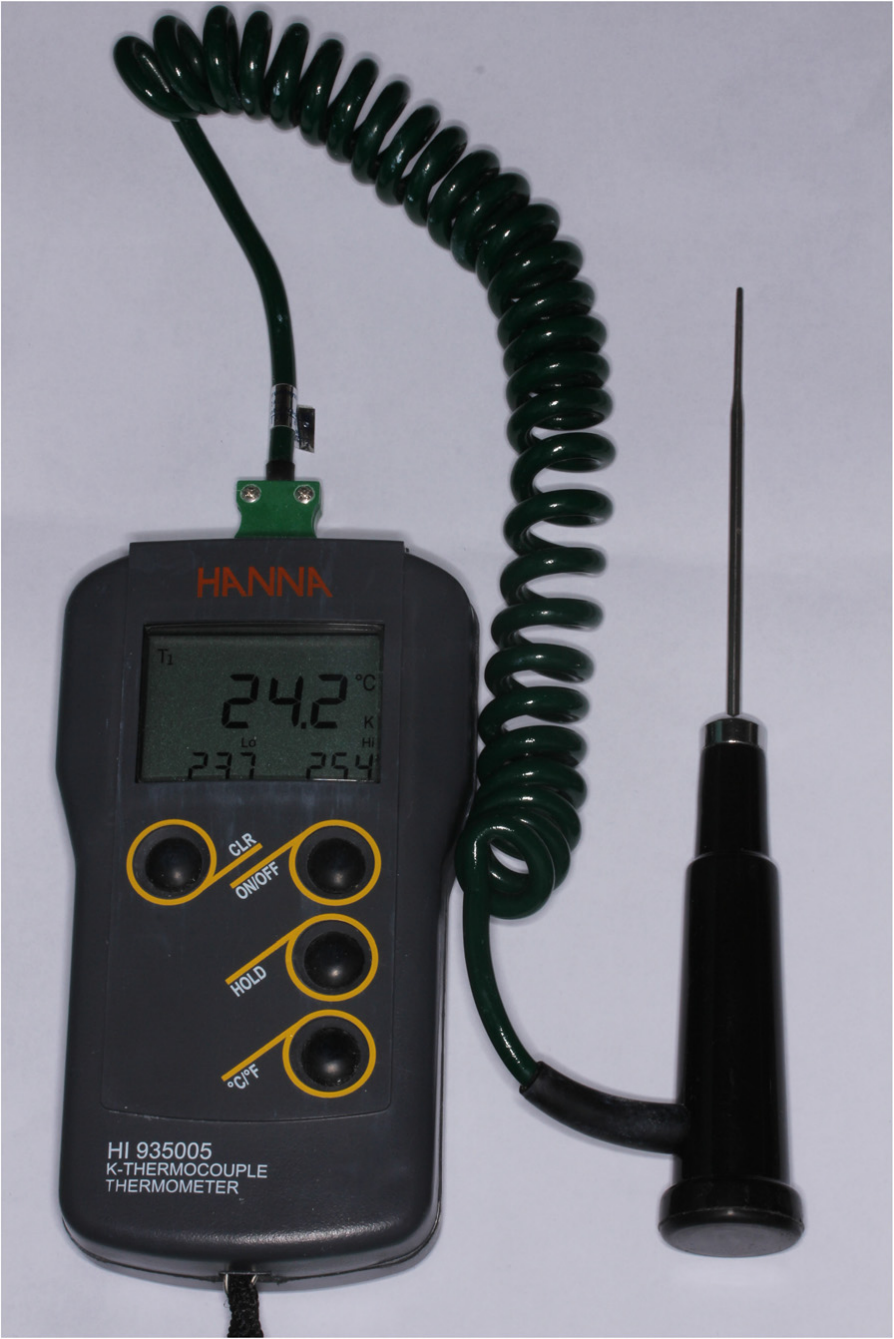
**J type Thermocouple connected to a digital thermometer.**

Thereafter, the pulp chamber was slightly dried and filled with a silicon heat transfer composite (Philips ECG Inc., Waltham, MA, USA) to facilitate heat transfer. A J type thermocouple with 1.60 mm diameter (J Type Thermocouple HI 766 series, Hanna, São Paulo, Brazil) was inserted into the pulp chamber connected to a digital thermometer to measure the temperature (Figure 1).

**Figure 2:**
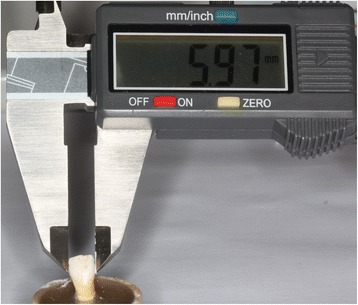
**Initial measurement of the tooth.**

**Figure 3:**
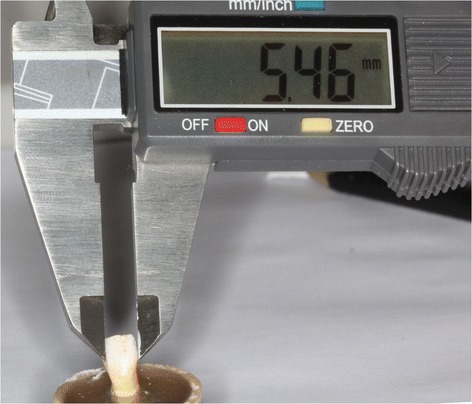
**Definition of the amount of stripping.**

The teeth were randomly divided per side, in order to perform a 0.5-mm stripping of interproximal enamel of the same tooth by a different technique. The amount of stripping was established by using a digital caliper on the buccal surface of each surface of the tooth (Figures 2 and 3).

After the stripping of every five teeth, the perforated stripping disks were replaced, and the metal handheld strippers were changed after every surface worn.

The stripping was performed by a single operator with the techniques described as follows:

PSD group: the stripping was performed using double-faced perforated stripping disks (7016, KG Sorensen, São Paulo, Brazil) without refrigeration.

HS group: the stripping was performed using 6-mm metal handheld strippers (3812.8271, KG Sorensen, São Paulo, Brazil).

**Figure 4:**
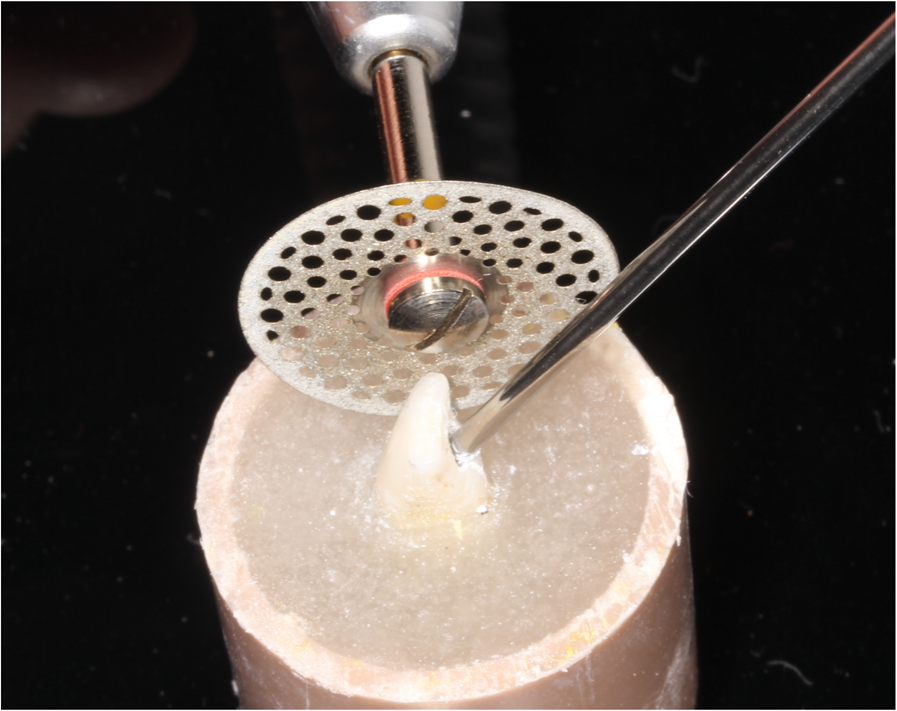
**Disk and thermocouple positioned for the procedure.**

The stripping procedure with PSD is presented in Figure 4 and the same sequence was performed with HS group but with use of handheld strippers.

The minimum and maximum temperatures produced, during the stripping process, were recorded on the thermometer display. The subtraction of the maximum temperature to the minimum temperature determined the temperature range throughout the procedure. The thermometer display was covered during the procedure and the temperature revealed only after all the stripping had already been performed.

The data obtained from all measurements were processed with SPSS 20 software, and the statistical analysis was performed through the analysis of variance test (ANOVA) followed by the Tukey's honestly significant difference (HSD) *post hoc* test, which verified whether significant difference existed or not between the averages and whether the factors exert some influence on any dependent variable. The level of significance was set at *p* < 0.005.

## Results

The descriptive statistics for each experimental group is shown in Table 1. ANOVA and *post hoc* test showed that the temperature of the pulp chamber varied significantly depending on the grinding procedure used (*p* < 0.001), being higher for the perforated stripping disk group, but there was no significant difference for the temperature variation between the three groups of teeth.

**Table 1:** **Descriptive statistics and ANOVA/Tukey comparison between the groups in relation to the rise in the temperature**

	**Number**	**Greater temperature rise (°C** **)**	**Average temperature rise (°C** **)**	**Standard deviation (°C** **)**	***p***	
Incisor PSD	13	3.1	2.58	0.27	A	<0.001
Incisor HS	13	1.7	1.24	0.3	B	
Premolar PSD	13	3.1	2.64	0.29	A	<0.001
Premolar HS	13	1.9	0.96	0.39	B	
Molar PSD	13	3.2	2.48	0.38	A	<0.001
Molar HS	13	1.2	0.92	0.18	B	

Different letters indicate statistical difference. PSD, perforated stripping disk; HS, handheld stripper.

**Figure 5:**
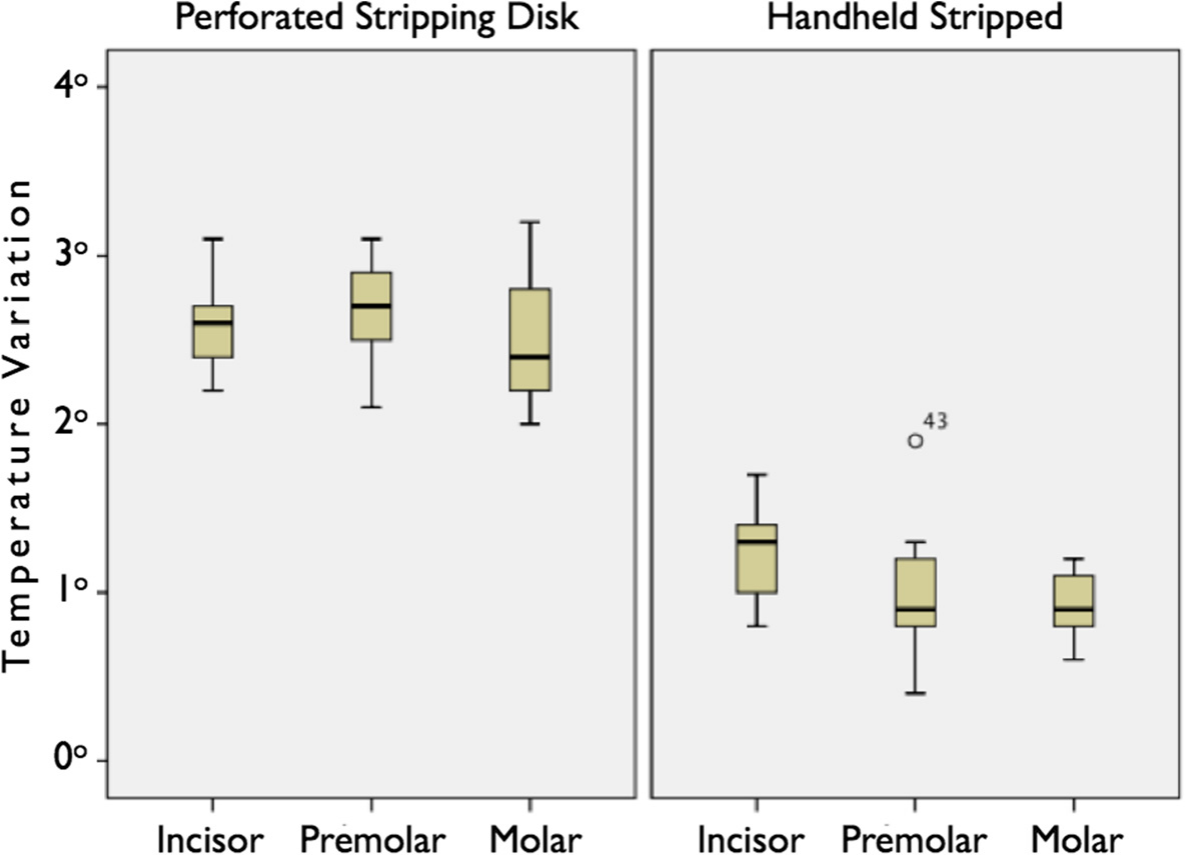
**Graph of the instruments (disk/stripper) and groups of teeth in relation to temperature variation.**

Figure 5 shows the temperature variation dividing the groups according to the instrument and tooth type. Besides, it is observed in Table 1 that the highest temperature was produced by perforated stripping disks in molars (3.2°C) followed by 3.1°C in premolars and incisors.

## Discussion

Taking into account that the use of instruments for stripping tooth enamel can cause irreversible changes in the pulp tissue by the rise of temperature, this *in vitro* study aimed to evaluate the change in temperature of the pulp chamber during stripping with metal handheld stripper and with perforated stripping disk at low speed, both techniques without refrigeration to enhance visualization of the operative field.

The thermocouple unit was selected to evaluate the pulp chamber temperature change because of its high accuracy and reliability of the results according to previous studies [15-19]. Thermal changes on the pulp tissue were evaluated in the teeth which were subjected to different types of procedures such as dental cavities, resin composite light curing, laser application, orthodontic bonding and debonding, and stripping in several studies in the areas of orthodontics and restorative dentistry [2,15,19].

The present study used intact human teeth: 13 lower incisors, 13 premolars, and 13 molars, totaling 78 surfaces that were worn. Theoretically, by presenting a higher risk of thermal damage due to the thin thickness of enamel and dentin, besides being the tooth group which most frequently undergoes this procedure [20], the implications of stripping on the lower incisors have significant importance for the clinical practice of orthodontics.

As a way to standardize the stripping procedure, some studies used the time or the number of applications of the metal handheld stripper on the teeth [15]. In the present study, it was decided to limit to 0.5 mm the amount of stripping on the enamel, measured with the aid of a digital caliper (Digimess, São Paulo, Brazil). Each surface of the teeth was stripped continuously until the removal of the enamel portion specified in order to determine the temperature variation during the performance of both stripping techniques.

According to the classic study by Zach and Cohen [1], pulp temperature rising greater than 5.5°C resulted in irreversible inflammation in 40% of the specimens evaluated. Moreover, according to these authors, increasing dental pulp temperature above 11°C invariably results in pulp necrosis. In the present study, an increasing pulp temperature was observed in all groups; however, the critical threshold of 5.5°C increase was not reached.

The stripping with perforated stripping disks produced a higher rise in the temperature than with metal handheld strippers. Comparing the groups of teeth, there was no difference in the temperature variation. This fact contradicts what was expected; the thickness of the enamel and the dentin of molars did not prevent them from suffering temperature increase. One possible explanation for this finding would be due to the higher buccal-lingual volume of the enamel found in molars, requiring greater amount of time for removal of the same tissue thickness.

A similar study found safe values for most types of stripping procedures with metal handheld strippers and perforated stripping disks at low speed. However, the threshold value of 5.5°C was reached in some cases, demonstrating the need for care during the procedure [15]. The greatest variation in recorded temperature during this study was 3.2°C when using perforated stripping disks in molars, therefore, below the critical value. The explanation for greater temperature rise in the Baysal et al. [15] study is the fact that they have used 10 s on each surface to perform the stripping, while our study used 0.5 mm, independent of time. Drills of high speed require refrigeration and do not promote significant increase in the temperature of the pulp. However, in this study, only techniques that do not require refrigeration were used.

As Raab [21] and Uysal et al. [19], this *in vitro* experimental study did not consider the dissipation of heat inside the pulp chamber due to the effect of blood circulation and fluid movement in the dentinal tubules. On the other hand, the pulp temperature increase may be clinically greater in young teeth because of the smaller thickness when compared with the dentin present in adult teeth in which the deposition of secondary dentin is enhanced [21].

## Conclusions

Considering the results obtained in this study, it can be concluded that

- Both stripping techniques produced an increase in temperature in the pulp chamber; however, this increase did not reach the critical value of 5.5°C with either technique.
- The perforated stripping disk produces higher increase in the temperature than the metal handheld strippers.
- There was no difference in temperature increase according to the group of teeth tested.
